# The Ambiguous Morpheme Processing in Chinese Compound Word Recognition in Deaf Readers

**DOI:** 10.3390/bs15121625

**Published:** 2025-11-25

**Authors:** Yang Liu, Mengfang Zhang, Yan Wu

**Affiliations:** 1 College of Computer Science and Technology, Changchun Normal University, Changchun 130032, China; liuyang@ccsfu.edu.cn; 2 Dalian High-Tech Zone No. 2 School, Dalian 116000, China; mfzhang901@nenu.edu.cn; 3 School of Psychology, Northeast Normal University, Changchun 130024, China

**Keywords:** ambiguous morpheme, word recognition, deaf, ERP

## Abstract

This study used event-related potentials (ERPs) to examine how deaf individuals process ambiguous morphemes during Chinese compound word recognition in a masked priming lexical decision paradigm. Ambiguous morphemes were classified as balanced or biased, and two experiments employed a 3 × 2 within-subject design. Each morpheme’s two meanings served as both primes and targets. The independent variables were prime type (meaning1 vs. meaning2 vs. unrelated) and target type (meaning1 vs. meaning2), with meaning1 being the dominant meaning and meaning2 being the subordinate meaning for biased morphemes. In the N250 (sublexical processing), balanced morphemes showed a main effect of prime type: any orthographically similar prime elicited priming. In the N400 (semantic processing), an interaction of prime and target type emerged, with only contextually congruent meanings activated. For biased morphemes, interactions were observed across N250 and N400 stages. The dominant meaning was consistently activated: when the target was dominant, both meanings showed priming; when the target was subordinate, only the subordinate meaning produced priming. These results reveal a dissociation in how deaf readers process ambiguous morphemes: balanced morphemes rely on contextual information, whereas biased morphemes are influenced by meaning frequency. The findings provide novel insights into the temporal dynamics of morpheme-based lexical access in deaf Chinese readers, with implications for reading and vocabulary instruction.

## 1. Introduction

Morphemes are considered the smallest linguistic units that simultaneously carry orthographic and semantic information, serving as the fundamental building units of words (Bae et al., 2024; Voga & Giraudo, 2017). For instance, the Chinese compound “月光” (yue guang, moonlight) consists of Yue (“moon”) and Guang (“light”), each bearing independent meaning (H. Chen et al., 2024). A substantial body of research demonstrates that the processing of polymorphemic words involves morphemic decomposition, a process referred to as morpheme processing (Amenta & Crepaldi, 2012; Beyersmann et al., 2016; de Zubicaray, 2025; Rastle et al., 2004; Yablonski et al., 2019). Evidence also indicates that morphemic activation plays a critical role in Chinese word processing (L. Chen et al., 2025; Feng et al., 2025; Tsang & Chen, 2013b; Wu et al., 2017; S. Zhao et al., 2021). In Chinese, a single character usually corresponds to one morpheme and more than 72% of modern words are disyllabic compounds composed of morphemes (Liu et al., 2007). However, the mapping between characters and morphemes is not always one-to-one. For example, “月, yue” denotes the celestial moon in “月光, yue guang” but refers to the temporal unit “month” in “一月, yi yue” (January). This phenomenon, known as morphemic ambiguity (H. Chen et al., 2024; Taft & Nguyen-Hoan, 2010), is more prevalent in Chinese than in alphabetic languages such as English (L. Chen et al., 2025). Indeed, over 65% of Chinese characters are polysemous, carrying two or more meanings (Liu et al., 2007). Such extensive ambiguity provides a natural linguistic resource for examining morpheme processing and raises a central question: during word recognition, are multiple meanings of an ambiguous morpheme activated, and if so, when?

Two key factors may play an important role in the processing of ambiguous morphemes: semantic frequency and contextual information (L. Chen et al., 2025; Xu & Chen, 2025). Semantic frequency highlights the activation advantage of more frequent meanings, whereas contextual support emphasizes the role of linguistic context in guiding meaning activation and/or selection. Normally, ambiguous morphemes can be classified into two types based on the frequency of various meanings. One type could be classified as the balanced ambiguous morphemes, whose meanings occur with comparable frequency (H. Chen et al., 2024). And the others are biased ambiguous morphemes, in which one dominant meaning occurs far more frequently than the others (Tsang & Chen, 2013b; Wu et al., 2020).

Theoretical models differ in how they conceptualize the roles of frequency and context in ambiguous morpheme processing. The lemma model (Taft & Nguyen-Hoan, 2010) posits an abstract lemma level between surface orthography and functional representation. At this level, morphemic decomposition provides pre-activation for all potential meanings of an ambiguous morpheme (Tsang & Chen, 2013a). Each meaning is represented by a separate lemma, and its activation strength is positively correlated with its token frequency. Thus, dominant meanings enjoy higher activation levels and lower thresholds, giving them a processing advantage and potentially suppressing subordinate meanings. In contrast, balanced morphemes activate their meanings with comparable strength, leading to competition among interpretations. However, the multiple access model proposes that all meanings of an ambiguous morpheme are initially activated equally, irrespective of frequency, and that context subsequently guides meaning selection (Kintsch & Mross, 1985; Van Petten & Kutas, 1987). Within this framework, two competing accounts of activation time course have been advanced: the form-then-meaning account, which assumes morphemic form is activated before meaning (Lavric et al., 2012; Morris & Stockall, 2012; Rastle & Davis, 2008; Wang et al., 2024), and the form-with-meaning account, which argues for parallel activation of form and meaning (Feldman et al., 2009; Reznick & Friedmann, 2015; Tsang & Chen, 2014; Wu et al., 2020).

Empirically, each perspective has its own supporting evidence. Using masked and unmasked priming paradigms, Tsang and Chen (2013b) demonstrated that for biased morphemes, primes corresponding to subordinate meanings facilitated recognition of dominant targets only under masked conditions; this effect disappeared when primes were unmasked. This pattern suggests that at early processing stages, when contextual cues are weak, the dominant meaning—with its lower activation threshold—can be triggered even by mismatched primes, thereby facilitating recognition. This is consistent with the frequency-driven mechanism proposed by the lemma model (Taft & Nguyen-Hoan, 2010). In contrast, no facilitation from subordinate primes was observed for balanced morphemes, and under unmasked conditions, slight inhibition even emerged. Such results likely reflect strong competition between meanings of comparable activation strength, which reduces the recognition benefit of shared morphemic form.

Complementary ERP evidence further clarifies the temporal dynamics. S. M. Zhao et al. (2017) reported that for the balanced morphemes, facilitation effects appeared at the N250 time window when primes and targets shared the same morpheme meaning, and these effects also evoked the changes of N400. These findings support a form-with-meaning activation mode and underscore the early influence of context on morphemic activation. Building on this line of research, priming paradigms combined with ERP techniques have been widely employed to investigate the processing of ambiguous words (Cai et al., 2023; Guo & Yilizhati, 2025; Wu et al., 2020; Wang et al., 2024). In general, two ERP components are most frequently examined in studies of morpheme processing: the N250 and the N400. The N250 is typically associated with early sublexical processing, including sub-lexical form-phonology and form-meaning mappings (S. M. Zhao et al., 2017; Wu et al., 2020; Guo & Yilizhati, 2025). In contrast, the N400 reflects semantic integration at both the morphemic and whole-word levels (Guo & Yilizhati, 2025; Wang et al., 2024).

Importantly, however, these findings are derived exclusively from hearing populations. For deaf and hard-of-hearing individuals (hereafter, “deaf individuals”), the processing of ambiguous morphemes remains largely unexplored. Deaf individuals experience impaired or absent auditory perception due to structural or functional abnormalities in the auditory system (Fang & Lei, 2015). The sensory/motor model posits that sensory and motor systems play a foundational role in constructing semantic knowledge, and that semantic representations are grounded in perceptual experience, including visual, auditory, and motor modalities (Martin et al., 2007). From this perspective, the absence of auditory input in deaf individuals is expected to alter the formation, storage, and retrieval of semantic information. Empirical evidence from lexical–semantic judgment tasks indeed demonstrates that both deaf children and deaf adults show reduced proficiency in semantic processing compared with their hearing peers (McEvoy et al., 1999; Ormel et al., 2010).

According to the sensory/motor model, morphemic semantic processing in deaf individuals is likely to be affected. Such effects may manifest either as a reduced ability to effectively extract morphemic meaning to facilitate whole-word semantic access, or as a delayed engagement of morphemic semantics during processing. This raises an important theoretical question: Do deaf readers possess lemma-level representations corresponding to morphemic meanings within their mental lexicon (Taft & Nguyen-Hoan, 2010)? If such representations exist, their temporal dynamics during word recognition remain to be clarified. This issue, in turn, also sheds light on the formation of lemmas.

Indeed, prior research on deaf individuals’ morphemic knowledge and decomposition skills paints a complex picture. On the one hand, even when matched for general language proficiency, deaf children lag by an average of five and a half years in morpheme knowledge relative to their hearing peers (Brown, 1984), and deaf adults still fall short of the level achieved by 9- to 10-year-old hearing children (Cooper, 1967). Deaf individuals also show pronounced deficits in morphemic decomposition: accuracy drops sharply when processing derived or low-frequency words, indicating generally weaker decomposition abilities than those of hearing individuals (Gaustad et al., 2002). Similarly, Van Hoogmoed et al. (2013) reported that deaf children rely significantly less on morphemic cues in lexical decision tasks, suggesting that reduced decomposition may further limit reading efficiency. Hearing students can effectively leverage morphemic decomposition as a decoding tool, whereas deaf students appear less able to decompose words into recognizable subunits and less adept at processing polymorphemic words (Cunningham & Allington, 1999).

On the other hand, some evidence suggests that limited phonological input may encourage deaf individuals to develop compensatory reliance on morpheme-based visual processing (Breadmore et al., 2012). Morphemic knowledge can aid recognition of longer words and support meaning construction (Sterne & Goswami, 2000). When morpheme knowledge reduces the difficulty of mapping orthography to semantics, gains in both word reading and comprehension are possible (Tong et al., 2011). For example, Breadmore et al. (2012) found that deaf children applied morphological rules in spelling regular and semi-regular plural nouns. Gaustad (2000) proposed a three-stage model of vocabulary development in deaf children, emphasizing the role of morpheme knowledge: initially, children learn words holistically through visual memorization; subsequently, they recognize morphemic components and use them to analyze longer words; finally, they integrate visual and morphemic structure analysis, gradually establishing a robust morpheme-based processing system. Thus, despite general delays in morpheme knowledge and decomposition skills, deaf individuals may nonetheless construct compensatory, visually driven morpheme-based pathways for lexical processing.

In sum, whether deaf individuals can effectively utilize morphemic information during word recognition remains an unresolved question. Theoretically, competing models yield divergent predictions, whereas empirically, the limited findings remain contested. Moreover, current research on morphemic processing in deaf individuals has been primarily inferential, with limited direct empirical evidence. To address this gap, the present study employed a masked priming paradigm in combination with ERP methods to examine the processing of ambiguous morphemes in Chinese lexical recognition among deaf individuals, focusing on both balanced and biased morphemes. By manipulating the overlap of form and meaning of morphemes between primes and targets, the study aims to answer three questions: (1) Do deaf individuals access morphemic information during Chinese word recognition, and (2) do balanced versus biased morphemes differ in processing patterns? (3) What is the time course of semantic activation for ambiguous morphemes?

To this end, two experiments were conducted. Experiment 1 investigated balanced morphemes, and Experiment 2 examined biased morphemes. Each experiment employed a 3 × 2 within-subjects design, with prime type and target type as the two factors. If morphemic semantics contribute to word recognition, an interaction between prime type and target type should emerge, for instance, the priming effect from meaning1/dominant to meaning1/dominant should differ from that from meaning1/dominant to meaning2/subordinate. Otherwise, only a main effect of prime type would be expected. In addition, if morphemic semantic activation aligns with a form-with-meaning parallel mapping model, the interaction between prime type and target type should appear at the N250 time window; if not, it should emerge only at the later N400 time window. Furthermore, if semantic frequency exerts a dominant influence, as predicted by the lemma model (Tsang & Chen, 2013a; Wu et al., 2020), the dominant meaning of biased morphemes should show a processing advantage, whereas balanced morphemes should show no meaningful differences between meanings. By comparing activation patterns at the N250 and N400 stages across morpheme types, the present study aims to clarify how meaning frequency and contextual support jointly shape morphemic semantic processing in deaf individuals.

## 2. Experiment 1: Balanced Morphemes

### 2.1. Participants

Behavioral and ERP experiments were conducted independently with different participants. For the behavioral experiments, in total, thirty-four deaf undergraduates from a university in Changchun were recruited. Four participants with accuracy rates below 50% were excluded, resulting in 30 valid participants (14 females; *M*_age_ = 22.03 years, *SD* = 1.58). In the ERP experiment, 33 participants were initially recruited. However, four participants did not finish the experiment and two participants were excluded due to technical failures, leaving 27 valid datasets (18 females; *M*_age_ = 22.56 years, *SD* = 1.42).

All participants had severe-to-profound hearing loss (hearing threshold ≥ 80 dB HL) and did not use hearing aids or cochlear implants during the experiment to avoid potential interference with EEG recording. All parents of the deaf participants were hearing, and thus none of the participants were native signers. However, all participants reported good proficiency in Chinese Sign Language and primarily used it for peer communication. All participants were prelingually deaf (either congenitally deaf or having lost hearing before the age of three). They had normal or corrected-to-normal vision, reported no neurological or cognitive disorders, and were right-handed. Written informed consent was obtained prior to participation, and participants received monetary compensation for their involvement.

### 2.2. Materials

Experimental stimuli were adapted from the ambiguous morpheme database developed by S. M. Zhao et al. (2017). Morpheme selection followed a rigorous procedure. Candidate ambiguous morphemes were extracted from the latest edition of the Xinhua Dictionary. A Semantic Association Questionnaire was then administered to 45 undergraduates (not involved in the main study), who provided first-association meanings for each candidate morpheme. For inclusion, morphemes had to meet two criteria: (a) each meaning had to be reported between 20% and 65% of the time, and (b) the difference in frequency between meanings had to be less than 30%. This ensured that both meanings were relatively balanced in salience. The final morpheme set had a mean frequency difference of 0.14 (*SD* = 0.08), consistent with the natural distribution of Chinese morpheme usage.

To further validate semantic distinctiveness, 15 additional undergraduates rated the semantic relatedness of the two meanings of each morpheme on a 6-point scale (1 = completely unrelated, 6 = highly related). The mean of the distinctiveness score was 2.21 (*SD* = 0.52), confirming that the two meanings were clearly differentiated. Prime–target pairs were constructed for both meanings of each morpheme, with all items being two-character words and non-repetitive across the stimulus set.

As shown in Table 1, primes did not differ significantly across conditions in stroke number, character frequency, or word frequency (all *Fs* < 1.5, *ps* > 0.20). Similarly, the two kinds of targets were also matched in stroke number, character frequency and word frequency (*ts* < 1.2, *ps* > 0.20). Additionally, another group of 15 students evaluated the semantic transparency of the materials (i.e., to rate how much the target morphemes contributed to the whole-word meanings) using a 6-point scale (1 = highly transparent, 6 = highly opaque). Results showed no differences in semantic transparency among the three prime types, *F*(2, 238) = 1.43, *p* = 0.20, and the two target types were also well matched, *t*(119) = 1.33, *p* = 0.10.

A separate group of 37 participants rated the whole-word semantic relatedness of the prime–target pairs on a 5-point Likert scale (1 = strongest relatedness). When the target corresponded to Meaning1, prime words sharing Meaning1 showed the strongest whole-word relatedness with the target (Meaning1 prime vs. unrelated: *p* < 0.001; Meaning1 prime vs. Meaning2 prime: *p* < 0.001), whereas no difference was observed between Meaning2 primes and unrelated controls (*p* = 0.42). Conversely, when the target corresponded to Meaning2, Meaning2 primes showed the strongest relatedness (Meaning2 prime vs. unrelated: *p* < 0.001; Meaning2 prime vs. Meaning1 prime: *p* < 0.001), with no difference between Meaning1 primes and unrelated controls (*p* = 0.15). It was, in fact, not possible to equate whole-word semantic relatedness across conditions, as words sharing the same ambiguous morpheme meaning are inherently more related at the lexical level. The potential influence of this confound on the present findings will be addressed in the Discussion section.

A Latin-square design was used to generate three counterbalanced lists, with each participant completing 240 real-word trials (80 per condition). An additional 240 pseudoword trials were created by recombining the two characters of target words. Pseudoword primes were identical to those in the real-word trials, with two-thirds sharing a morpheme with the prime and one-third not sharing.

### 2.3. Procedure

**Figure 1:**
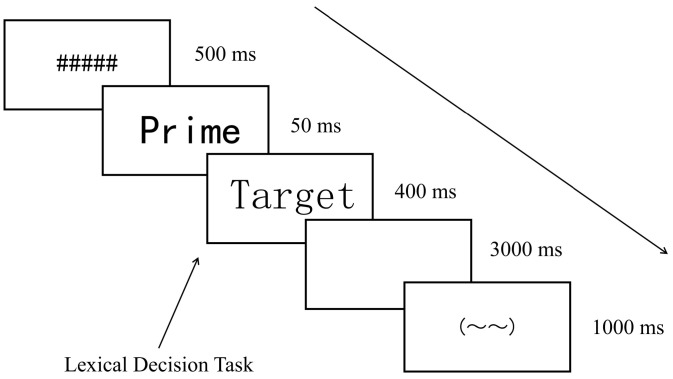
Diagram of the experimental procedure. “#####” indicate the mask, and “~~” is the rest cue.

The visual lexical task was implemented in E-Prime 3.0 and presented on a CRT monitor with a 60 Hz refresh rate (see Figure 1). Each trial began with a 500 ms random-line mask, followed by a 50 ms prime (KaiTi font, size 46). The target word (SongTi font, size 48) then appeared for 400 ms. Participants were required to judge whether the target was a real word. Half of the participants pressed “Z” for real words, while the other half pressed “M.” After the response or after 3000 ms with no response, a 1000 ms fixation cross was presented as a rest cue. To minimize visual confounds, primes and targets were displayed in different font styles and sizes.

Each version of the experiment contained 480 trials (240 real words, 240 pseudowords), divided into 10 blocks of 48 trials. Participants could rest between blocks at their own pace. A short practice session preceded the main task to ensure task familiarity. The entire experiment lasted approximately 1 h.

### 2.4. Data Recording and Analysis

Behavioral performance was assessed using accuracy (ACC) and reaction time (RT). Accuracy was coded as 1 for correct and 0 for incorrect responses. RT data were analyzed using linear mixed-effects models (LMMs; lme4 (v 1.1 -36) package, R), while accuracy data were analyzed using logistic regression, which is appropriate for binary outcomes. Linear mixed-effects models were constructed with accuracy and RTs as dependent variables, prime type and target type as a fixed effect, and participants and items as random effects.

EEG data were recorded using a 64-channel Neuroscan 4.3 system with Ag/AgCl electrodes, positioned according to the international 10–20 system. The left mastoid served as the reference, with the right mastoid also recorded. Horizontal and vertical EOGs were recorded, and the ground electrode was placed at the midpoint between FPz and Fz. Electrode impedances were maintained below 5 kΩ. EEG signals were sampled at 1000 Hz and filtered online from 0.05 to 100 Hz.

Offline processing was conducted using the EEGLAB toolbox (v2023.1). Data were re-referenced to the average of both mastoids and filtered from 0.05 to 30 Hz via the pop_eegfiltnew function. Epochs were extracted from −200 ms to 1000 ms relative to target onset, with the −200 to 0 ms interval used for baseline correction. Trials contaminated by ocular or muscular artifacts, or exceeding ±100 µV, were excluded.

Based on prior research (Cao et al., 2022; Wu et al., 2020), the N250 was analyzed in the 200–300 ms time window, N400 was analyzed in the 300–500 ms time window. Mean amplitudes for each time window were subjected to three-way repeated-measures ANOVAs with factors of Prime Type (meaning1, meaning2 and unrelated), Target Type (meaning1 and meaning2) and Scalp Region (F, FC, C, CP, P). Each region included three electrodes: F (F3, FZ, F4), FC (FC3, FCZ, FC4), C (C3, CZ, C4), CP (CP3, CPZ, CP4), and P (P3, PZ, P4). Greenhouse-Geisser corrections were applied where appropriate (Greenhouse & Geisser, 1959).

### 2.5. Results

#### 2.5.1. Behavioral Results

Table 2 presented the mean RTs and accuracy rate of the behavioral experiments. The best-fitting converged model included the main effect of prime type, target type, the interaction between prime type and target type and selected random effects for participants and items. For accuracy, the likelihood ratio test indicated that the converged model did not differ significantly from the null model (*χ*^2^ = 1.83, *df* = 5, *p* = 0.87). Further analysis on accuracy didn’t reveal any significant effect (*p*s > 0.45). For RT, after excluding trials with responses beyond ±2.5 *SD*s and incorrect responses (19.69%), the converged model did not differ from the null model (*χ*^2^ = 10.35, *df* = 5, *p* = 0.06). Further analysis of the RTs did not reveal any significant effects (*p*s > 0.10).

#### 2.5.2. ERP Results

**Figure 2:**
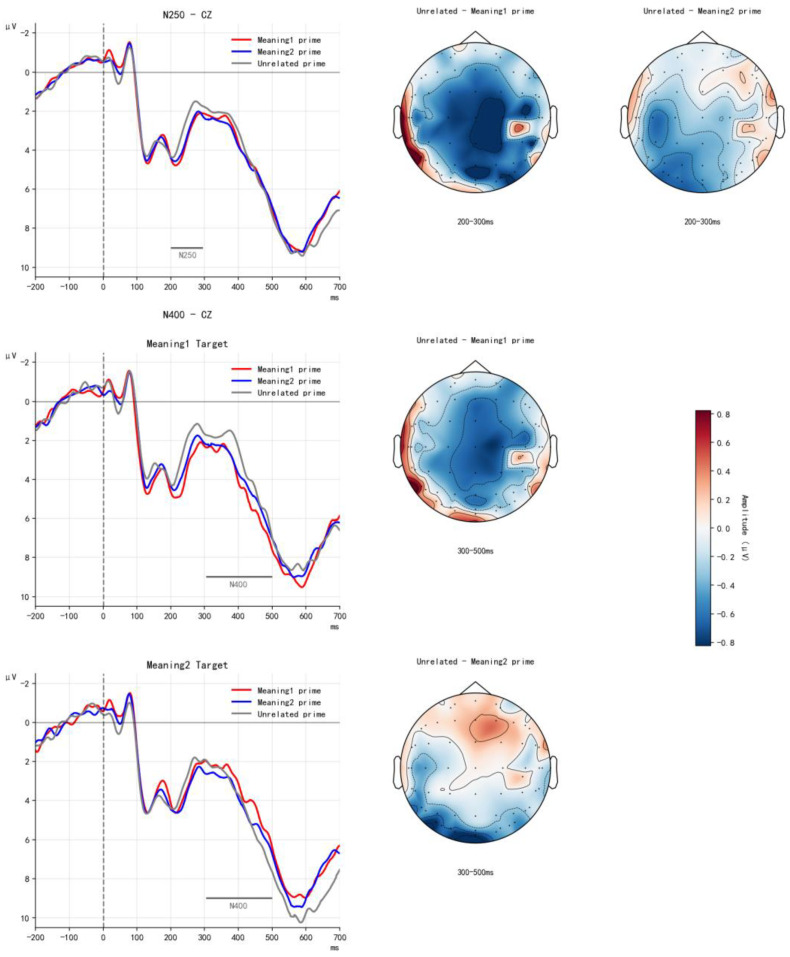
Grand-averaged waveforms for each condition in the balanced-morpheme experiment at Cz, along with their corresponding difference topographic maps. The upper panel illustrates the main effect of prime type during the N250 time window, whereas the lower panel depicts the interaction between prime type and target type during the N400 time window.

After excluding artifacts and incorrect responses, 24.4%, 26.8%, and 26.4% of trials were discarded for the meaning1 prime, meaning2 prime, and unrelated conditions, respectively, when meaning1 served as the target. Under the meaning2 target condition, the proportions of discarded trials were 26.5%, 21.3%, and 23.8%, respectively. The relative high trial rejection rate resulted from the relatively low accuracy of lexical decision performance in deaf participants. The accuracy rate in the ERP experiment ranged from 0.84 to 0.90 across participants. Figure 2 presents the grand-averaged waveforms for each condition in the balanced-morpheme experiment, along with the corresponding difference topographic maps across the two time windows.

##### 200–300 ms Time Window

A significant main effect of prime type was observed, *F*(1.87, 84.74) = 4.75, *p* = 0.015, η^2^ₚ = 0.15. Follow-up comparisons showed that the N250 elicited by the meaning1 prime was smaller than that elicited by the unrelated prime (β = −0.51, *SE* = 0.18, *t* = −2.69, *p* = 0.032). The difference between the meaning2 prime and the unrelated condition was also significant (β = −0.49, *SE* = 0.20, *t* = −2.54, *p* = 0.043). No difference was found between the meaning1 and meaning2 primes (β = 0.017, *SE* = 0.16, *t* = 0.11, *p* = 0.99). No other effects reached significance (*p*s > 0.30). These results indicate that, for target words containing balanced morphemes, early processing is facilitated as long as the prime and target share morphemic form.

##### 300–500 ms Time Window

A significant interaction between prime type and target type was found, *F*(1.64, 42.73) = 5.22, *p* = 0.013, η^2^ₚ = 0.17. Follow-up analyses showed that for meaning1 targets, the meaning1 prime elicited a smaller N400 than the unrelated control (β = 0.65, *SE* = 0.23, *t* = 2.82, *p* = 0.02). No significant differences were observed for meaning2 targets (*p*s > 0.10). These findings suggest that in the processing of balanced morphemes, early facilitation arises from shared morphemic form, whereas in later semantic processing, facilitation occurs only when the prime and target share morphemic meaning.

## 3. Experiment 2: Biased Morphemes

### 3.1. Participants

The behavioral experiments for Experiment 2 and Experiment 1 were drawn from the same participant pool. Initially, 34 deaf college students were recruited. For the biased morpheme condition, one participant did not complete the task; thus, data from 33 participants were included in the analysis (17 females; *M*_age_ = 21.88 years, *SD* = 1.47). The ERP experiments for Experiment 2 and Experiment 1 also recruited from the same participant group. For the biased morpheme condition, five participants were excluded due to excessive EEG artifacts, and one was excluded due to a technical failure, yielding a final sample of 27 participants (17 females; *M*_age_ = 21.54 years, *SD* = 1.38). To avoid order effects, the sequence of the two behavioral experiments and the two ERP experiments was counterbalanced across participants.

### 3.2. Materials

The selection and compilation procedure for biased morphemes was identical to that for balanced morphemes, with the exception of the semantic frequency criteria. Specifically, to ensure that each selected ambiguous morpheme had a clearly defined dominant and subordinate meaning, the following criteria were applied: the dominant meaning had to be reported in more than 65% of cases, the subordinate meaning in fewer than 20% of cases, and the frequency difference between the two meanings had to exceed 60%. A total of 120 ambiguous morphemes meeting these criteria were selected. For these morphemes, the mean frequency difference between dominant and subordinate meanings was 0.76 (*SD* = 0.11), and the average semantic distinctiveness rating was 2.2 (*SD* = 0.49).

Prime words did not differ significantly in stroke number, mean character frequency, or word frequency across conditions (*Fs* < 1.77, *ps* > 0.10). Likewise, target words showed no significant differences on these measures (*ts* < 1.63, *ps* > 0.10) (see Table 3). A rating test was completed by 18 participants who did not take part in other tests. They were asked to rate how much the target morphemes contributed to the whole-word meanings on a 7-point Likert scale (7 = strongest contribution). There were no significant differences in transparency ratings among the three priming conditions (*F*(2, 238) = 1.06, *p* = 0.357) or between the two target conditions (*t*(119) = 0.83, *p* = 0.419).

In addition, another group of 36 participants rated the whole-word semantic relatedness of the prime–target pairs on a 5-point Likert scale (1 = strongest relatedness). The results showed that when the dominant meaning served as the target, the dominant-meaning prime was rated as most semantically related to the target (dominant prime vs. unrelated: *p* < 0.001; dominant prime vs. subordinate prime: *p* < 0.001). No difference was observed between the subordinate-meaning prime and the unrelated control (*p* = 0.23). When the subordinate meaning served as the target, the subordinate-meaning prime was rated as most semantically related to the target (subordinate prime vs. unrelated: *p* < 0.001; subordinate prime vs. dominant prime: *p* < 0.001). No difference was found between the dominant-meaning prime and the unrelated control (*p* = 0.38). It was not possible to equate whole-word semantic relatedness across conditions because words sharing an ambiguous morpheme in the same meaning are inherently more related at the whole-word level. The potential impact of this confusion on the present findings will be addressed in the Discussion section.

### 3.3. Procedure

Stimulus presentation and the experimental procedure was identical to that in the balanced morpheme experiment.

### 3.4. Data Recording and Analysis

EEG recording and preprocessing procedures were the same as those used in the balanced morpheme experiment. Trials contaminated by ocular or muscular artifacts, or exceeding ±100 µV, were excluded. Mean amplitudes for each time window were subjected to three-way repeated-measures ANOVAs with factors of Prime Type (dominant prime, subordinate prime vs. unrelated), Target Type (dominant target vs. subordinate target) and Scalp Region (F, FC, C, CP, P).

### 3.5. Results

#### 3.5.1. Behavioral Results

Table 2 presented the mean RTs and accuracy rate. For both accuracy and RTs, the best-fitting models included the main effect of prime type, target type, the interaction between prime type and target type and random effects for items and participants. For accuracy, model comparisons revealed significant difference between the final and the null models (*χ*^2^ = 11.56, *df* = 5, *p* = 0.041). Further analysis revealed the significant main effect of prime type (*χ*^2^ = 6.17, *df* = 2, *p* = 0.045). The accuracy rate for the subordinate prime was a little higher than unrelated condition (0.83 vs. 0.80, β = 0.27, *SE* = 0.11, *t* = −2.39, *p* = 0.04).

For RTs, trials with incorrect responses and those exceeding ±2.5 *SD*s from the mean were excluded (dominant target: 16.46%; subordinate target: 22.83%), and the final model fits significantly better than the null model (*χ*^2^ = 20.93, *df* = 5, *p* < 0.001). A significant interaction between prime type and target type was observed for RTs (*F*(2, 331.8) = 5.99, *p* = 0.003). Simple effect analysis revealed the dominate prime facilitate the processing of the dominant target (dominant prime vs. unrelated condition (β = 24.92, *SE* = 8.30, *t* = −3.00, *p* = 0.008); and the subordinate prime facilitate the processing of the subordinate target (subordinate prime vs. unrelated condition: β = 28.08, *SE* = 9.10, *t* = −3.08, *p* = 0.006; subordinate prime vs. dominant prime: β = 22.04, *SE* = 8.18, *t* = −2.69, *p* = 0.019).

#### 3.5.2. ERP Results

**Figure 3:**
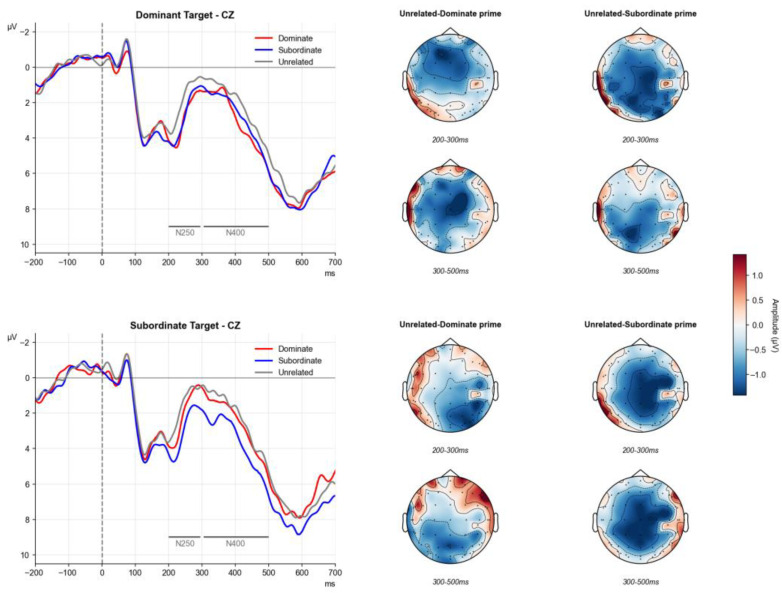
Grand-averaged waveforms of each condition for the dominant meaning target (**upper panel**) and subordinate meaning target (**lower panel**) at CZ and their corresponding difference topographic maps illustrating N250 and N400 effects.

Approximately 23.7%, 26.6%, and 26.6% of trials were excluded from the final analyses for the dominant prime, subordinate prime, and unrelated conditions, respectively, when the target corresponded to the dominant meaning. For the subordinate-meaning target, 33.1%, 28.0%, and 35.2% of trials were removed, respectively. The elevated trial rejection rate resulted from the relatively low accuracy of lexical decision performance in deaf participants. The accuracy rate in the ERP experiment ranged from 0.77 to 0.83 across participants. Figure 3 presents the grand-averaged waveforms for each condition under the dominant and subordinate meaning contexts, along with the corresponding topographic maps across the two time windows.

##### 200–300 ms Time Window

The three-way interaction among prime type, target type, and region was significant, *F*(3.05, 79.34) = 4.75, *p* = 0.004, η^2^_p_ = 0.155. Simple effect analyses revealed that for the dominant target, both the dominant and subordinate primes elicited smaller N250 amplitudes than the unrelated control in the C region (dominant vs. unrelated: β = 0.78, *SE* = 0.22, *t* = 3.44, *p* = 0.005; subordinate vs. unrelated: β = 0.78, *SE* = 0.25, *t* = 3.03, *p* = 0.014). Additionally, in the frontal (F) and fronto-central (FC) regions, the difference between the dominant prime and the unrelated condition was significant (F: β = 0.87, *SE* = 0.25, *t* = 3.43, *p* = 0.006; FC: β = 0.95, *SE* = 0.26, *t* = 3.71, *p* = 0.003), while in the centro-parietal (CP) and parietal (P) regions, the subordinate prime elicited significantly smaller N250 than the unrelated condition (CP: β = 0.83, *SE* = 0.24, *t* = 3.45, *p* = 0.005; P: β = 0.62, *SE* = 0.18, *t* = 3.42, *p* = 0.006). These findings indicate that for the dominant target, both dominant and subordinate primes facilitated early processing.

For the subordinate target, only the subordinate prime evoked a smaller N250 than the unrelated condition across FC, C, CP, and P regions (FC: β = 0.95, *SE* = 0.35, *t* = 2.68, *p* = 0.032; C: β = 0.96, *SE* = 0.27, *t* = 3.48, *p* = 0.005; CP: β = 1.11, *SE* = 0.28, *t* = 3.94, *p* = 0.002; P: β = 0.73, *SE* = 0.21, *t* = 3.51, *p* = 0.005), suggesting that early facilitation occurs only when the prime and target meanings match.

##### 300–500 ms Time Window

The three-way interaction among prime type, target type, and region was significant, *F*(3.23, 84.05) = 2.66, *p* = 0.049, η^2^_p_ = 0.09. For the dominant target, both dominant and subordinate primes elicited smaller N400 amplitudes than the unrelated control in the C region (dominant vs. unrelated: β = 0.53, *SE* = 0.19, *t* = 2.71, *p* = 0.003; subordinate vs. unrelated: β = 0.56, *SE* = 0.22, *t* = 2.50, *p* = 0.048), and in the CP and P regions, the subordinate prime was also significantly smaller than the unrelated condition (CP: β = 0.71, *SE* = 0.21, *t* = 3.30, *p* = 0.008; P: β = 0.53, *SE* = 0.19, *t* = 2.72, *p* = 0.029).

For the subordinate target, only the subordinate prime elicited smaller N400 amplitudes than the unrelated control across FC, C, CP, and P regions (FC: β = 1.01, *SE* = 0.27, *t* = 3.69, *p* = 0.003; C: β = 1.00, *SE* = 0.29, *t* = 3.44, *p* = 0.005; CP: β = 1.16, *SE* = 0.33, *t* = 3.54, *p* = 0.004; P: β = 0.72, *SE* = 0.21, *t* = 3.30, *p* = 0.008). Moreover, in the FC region, the N400 evoked by the subordinate prime was even smaller than that elicited by the dominant prime (β = 1.00, *SE* = 0.27, *t* = 3.61, *p* = 0.004). These results suggest that, for subordinate targets, only a meaning-matched prime facilitates later semantic processing.

## 4. Discussion

The present study combined a masked priming paradigm with ERP techniques to systematically examine how deaf adults process ambiguous morphemes in Chinese word recognition and to trace the temporal dynamics of this processing. By contrasting balanced and biased morphemes and manipulating the morphological relationship between primes and targets, we delineated the trajectory of morphemic activation. The results revealed that deaf participants accessed morphemic semantics in both conditions but followed different processing patterns. For balanced-morpheme targets, the two meanings competed, and no activation was observed during the early N250 stage; only in the N400 time window, reflecting semantic processing, did individual meanings become activated, highlighting the role of contextual selection. In contrast, for biased morphemes, the dominant meaning was activated at both early and later stages, regardless of whether the prime shared the same morphemic meaning, indicating that meaning frequency plays a critical role in morpheme processing. The subordinate meaning can also be activated, but only with contextual support, as reflected in the N250 and N400 effects.

Before considering the implications of the present findings, it is important to evaluate whether they truly reflect morphological processing. As noted in the Materials section, it was not possible to fully control for whole-word semantic relatedness. However, we argue that this potential confound is unlikely to have substantially influenced the results for three reasons. First, if whole-word semantic relatedness were driving the effects, words sharing both morphemic form and meaning would be expected to show the strongest priming. Yet, in the ERP data, this pattern did not emerge. In the balanced-morpheme condition, early processing was facilitated whenever primes and targets shared morphemic form, producing similar N250 effects despite differences in whole-word semantic relatedness. In the biased-morpheme condition, the dominant meaning was activated by both dominant and subordinate primes, as reflected in N250 and N400 amplitudes, again despite differing whole-word semantic associations. Second, previous research indicates that masked priming paradigms rarely produce whole-word semantic priming; even in ERP studies, word-level semantic effects typically require longer prime durations (e.g., 230 ms; Rastle et al., 2000; Wu et al., 2017). Third, prior studies using similar designs in hearing readers have reliably demonstrated morphological effects while effectively ruling out word-level semantic priming (Cai et al., 2023; Guo & Yilizhati, 2025; Wu et al., 2020; S. M. Zhao et al., 2017). Taken together, these considerations support the conclusion that the present results genuinely reflect morphological processing, with important theoretical and educational implications.

### 4.1. Distinctions Between Balanced and Biased Morpheme Processing in Deaf Readers

Morphemes play a central role in word recognition, yet whether deaf readers can reliably access morphemic information remains debated (Breadmore et al., 2012; Gaustad et al., 2002; Sterne & Goswami, 2000; Tong et al., 2011; Van Hoogmoed et al., 2013). Using a masked priming paradigm that minimizes conscious strategic processing, the present study demonstrated that deaf readers automatically activate morphemic semantics. Importantly, however, their processing patterns differed across morpheme types. For balanced morphemes, activation of morphemic semantics required contextual support and emerged only at the later N400 time window, indicating delayed, context-dependent semantic integration. In contrast, for biased morphemes, deaf readers exhibited a robust semantic frequency effect: even when context did not align with the target meaning—for instance, in the subordinate-prime to dominant-target condition—the dominant meaning was activated at both early and late stages, as evidenced by reliable N250 and N400 effects.

These findings partially diverge from patterns observed in hearing readers. S. M. Zhao et al. (2017) found that in hearing individuals, contextually congruent meanings of balanced morphemes can be activated very early, emerging in the N250 component. For biased morphemes, however, the present results align with hearing populations (Tsang & Chen, 2013b; Wu et al., 2020), consistently showing an activation advantage for the dominant meaning (Duffy et al., 1988). In cases where early linguistic experience is limited—such as with subordinate meanings—context exerts a determining influence. Yet, the late N400 effects for balanced morphemes in deaf readers cannot be fully explained by the sensory/motor model (Martin et al., 2007), since both dominant and subordinate meanings of biased morphemes were activated early. We propose that the delayed pattern for balanced morphemes primarily reflects competition between equally frequent meanings, which hinders efficient early activation and results in weaker late-stage effects, with only one meaning (meaning1) showing activation even when both were contextually supported. This competitive mechanism highlights that deaf readers are highly sensitive to morphemic semantic frequency, which plays a crucial role in their morphemic processing.

Overall, deaf readers can efficiently and rapidly extract morphemic semantics during word recognition, but this does not imply that auditory deprivation has no impact on semantic development. Their sensitivity to morphemic meanings likely reflects a visual compensation mechanism. Previous studies have shown that deaf readers compensate for absent or reduced phonological input by relying more heavily on visual information, exhibiting advantages in parafoveal processing (Qin et al., 2022), expanded perceptual span (Bélanger et al., 2012), and heightened sensitivity to visual word forms (Gutierrez-Sigut et al., 2022). Such adaptations facilitate direct form–meaning mapping, and in the absence of phonological support, the stable form–semantic correspondence characteristic of Chinese morphemes, combined with long-term reliance on written input, promotes enhanced sensitivity to morphemic semantics (Bélanger & Rayner, 2015; Thierfelder et al., 2020). Consequently, even subordinate meanings can be effectively activated when supported by context.

Sign language experience may further contribute to these effects. All participants were highly proficient signers who frequently used sign language in peer communication. Previous research has shown that sign language experience can facilitate written language processing in deaf individuals (Y. Zhao et al., 2020). In addition, sign language provides extensive semantic knowledge of nouns, verbs, adjectives, and other content words—the majority of its vocabulary—further supporting the extraction of morphemic semantics during word recognition.

### 4.2. Semantic Activation Dynamics of Ambiguous Morphemes in Deaf Readers

This study is the first to use ERP methods to examine the temporal dynamics of ambiguous morpheme processing in Chinese word recognition among deaf college students. The time course of morphemic processing has long been debated (Lavric et al., 2012; Morris & Stockall, 2012; Feldman et al., 2009; Reznick & Friedmann, 2015). The present findings add new evidence by showing distinct activation patterns for balanced and biased morphemes in a deaf population shaped by unique sensory and linguistic experiences.

For balanced morphemes, competition between equally frequent meanings appears to generate a form-then-meaning activation pattern. In contrast, biased morphemes exhibit a form-with-meaning pattern: both dominant and subordinate meanings can be activated at early processing stages, although the subordinate meaning requires contextual support whereas the dominant meaning does not. These results indicate that deaf readers can efficiently activate morphemic semantics, but the dynamics are modulated by meaning frequency and contextual constraints.

Taken together, the findings provide empirical evidence from a special population bearing directly on the ongoing debate between the Lemma model and multiple-access accounts. The Lemma model (Taft & Nguyen-Hoan, 2010) proposes that morphemes with different meaning frequencies show distinct activation profiles: balanced morphemes generate strong competition among lemmas, limiting form-based priming and making semantic consistency and contextual cues critical for meaning selection; dominant meanings, by contrast, have lower activation thresholds and can be triggered by form overlap alone. However, in contrast to the strict predictions of the Lemma model, the present results reveal that subordinate meanings may also be rapidly and effectively activated when meaning competition is relatively low and contextual information provides sufficient support. This pattern aligns with multiple-access models, which hold that context governs later selection processes (Kintsch & Mross, 1985; Van Petten & Kutas, 1987).

Overall, the processing of morphemic semantics in deaf readers appears to reflect a hybrid visual-lemma architecture: lemma-level semantic representations exist, but their activation is shaped first by experience-dependent adaptations to visual dominance and auditory deprivation, and then by contextual constraints. These findings extend the applicability of both the Lemma model and multiple-access accounts to special populations, underscoring the importance of incorporating experience-dependent variability into models of language processing (Glezer et al., 2018; Meade et al., 2020). They refine our understanding of how ambiguous morphemes are processed and offer a foundation for developing language models tailored to populations with atypical sensory or linguistic experience, in which meaning frequency and contextual support jointly serve as key mechanisms for resolving semantic ambiguity.

### 4.3. Educational Implications and Limitations

The present findings yield important pedagogical implications. Ambiguous words, which are highly prevalent in Chinese, pose considerable challenges for deaf learners due to the intricate mapping between orthographic form and semantic meaning. The current study elucidates how semantic frequency and contextual support differentially contribute to the processing of ambiguous morphemes, thereby offering evidence-based guidance for Chinese language instruction. In practice, teachers may present ambiguous characters within multisyllabic compounds structured according to their relative meaning dominance to enhance instructional effectiveness. Such compounds not only supply the contextual cues necessary for disambiguation but also facilitate the acquisition of balanced morphemes by providing stable and meaningful semantic environments.

It is worth noting, however, that this study relied solely on visually presented stimuli. In real communication, deaf individuals’ comprehension often draws on multiple channels, including sign language, lip-reading, facial expressions, and residual hearing. Future research should adopt multimodal input paradigms that better approximate naturalistic contexts, thereby clarifying the contribution of different perceptual channels. Moreover, reliance on multimodal cues may vary across subgroups of deaf readers. Those with greater oral language experience may depend more on lip-reading and auditory cues, whereas proficient signers may prioritize mappings between sign language and meaning (Lan et al., 2023; Lieberman et al., 2015). Integrating individual language experience into future designs will be crucial for advancing our understanding of multimodal integration in morpheme processing.

## 5. Conclusions

This study investigated how deaf individuals process ambiguous morphemes during Chinese compound word recognition using a masked priming lexical decision paradigm and event-related potentials (ERPs). The findings revealed distinct processing patterns for balanced and biased morphemes. These results provide important insights into the temporal dynamics of morpheme-based lexical access in Chinese and shed light on how deaf readers process ambiguous morphemes.

## Figures and Tables

**Table 1:** Examples and Properties of Balanced Morpheme Materials.

	Primes			Targets	
	Meaning1	Meaning2	Unrelated	Meaning1	Meaning2
Examples	公众 public	公鸡 rooster	补丁 patch	公共 public	公母 sex of animals
Stoke Number	16.03 (3.58)	16.19 (3.53)	15.68 (2.93)	15.73 (4.04)	15.63 (4.16)
Mean Character Frequency (log)	4.82 (0.48)	4.87 (0.45)	4.85 (0.37)	4.88 (0.47)	4.89 (0.44)
Semantic Transparency	3.20 (0.17)	3.35 (0.10)	3.43 (0.09)	3.42 (0.09)	3.58 (0.08)
Word Frequency (log)	3.12 (0.89)	3.16 (0.87)	3.22 (0.51)	3.45 (0.89)	3.32 (0.92)
Word semantic relatedness with meaning1 target	2.55 (0.68)	3.60 (0.71)	3.69 (0.91)		
Word semantic relatedness with meaning2 target	4.13 (0.49)	2.66 (0.81)	4.21 (0.47)		

Note. Values in parentheses represent standard deviations. Character frequency was log-transformed (base 10) and averaged across constituent characters. Word frequency norms were obtained from the Center for Chinese Linguistics, Peking University, and character frequency norms were based on the Modern Chinese Single-Character Frequency List.

**Table 2:** Mean reaction time and accuracy rate for the two behavioral experiments.

Experiment	Target Type	Prime Type	RT (ms)	ACC
Balanced Morphemes	Meaning1	Meaning1	543.53 (128.37)	0.84 (0.13)
		Meaning2	556.88 (132.35)	0.81 (0.13)
		Unrelated	578.54 (128.84)	0.82 (0.14)
	Meaning2	Meaning1	569.62 (135.14)	0.84 (0.12)
		Meaning2	546.34 (123.90)	0.82 (0.12)
		Unrelated	580.93 (132.34)	0.82 (0.16)
Biased Morphemes	Dominant	Dominant	577.36 (156.26)	0.85 (0.11)
		Subordinate	591.35 (161.99)	0.86 (0.11)
		Unrelated	603.14 (164.57)	0.83 (0.13)
	Subordinate	Dominant	603.81 (176.32)	0.79 (0.15)
		Subordinate	581.77 (161.81)	0.80 (0.12)
		Unrelated	607.01 (171.81)	0.78 (0.14)

**Table 3:** Examples and Properties of Biased Morpheme Materials.

	Primes			Targets	
	Dominant	Subordinate	Unrelated	Dominant	Subordinate
Examples	批评 criticize	批发 wholesale	春耕 spring plowing	批改 correct	批购 purchase in bulk
Stoke Number	16.33 (4.25)	16.88 (4.10)	16.52 (2.94)	16.59 (4.19)	16.80 (4.39)
Mean Character Frequency (log)	4.79 (0.51)	4.83 (0.48)	4.76 (0.42)	4.83 (0.43)	4.83 (0.48)
Semantic Transparency	4.79 (0.71)	4.87 (0.57)	4.63 (0.60)	4.60 (0.54)	4.42 (0.54)
Word Frequency (log)	6.80 (0.004)	6.80 (0.016)	6.80 (0.004)	6.80 (0.004)	6.80 (0.012)
Word semantic relatedness with dominant target	2.58 (0.71)	3.90 (0.61)	4.01 (0.65)		
Word semantic relatedness with subordinate target	3.97 (0.68)	2.79 (0.71)	4.04 (0.45)		

Note. Values in parentheses represent standard deviations. Character frequency was log-transformed (base 10) and averaged across constituent characters. Word frequency norms were obtained from the Center for Chinese Linguistics, Peking University, and character frequency norms were based on the Modern Chinese Single-Character Frequency List.

## Data Availability

The data presented in this study are available on request from the corresponding author. The data are not publicly available due to privacy.
